# Violations of Coordination: Exploring Metastable Diborides via Energetic Transition Metals

**DOI:** 10.1021/jacs.5c04066

**Published:** 2025-05-02

**Authors:** Joseph T. Doane, Gregory M. John, Alma Kolakji, Abraham A. Rosenberg, Yiren Zhang, Alan A. Chen, Michael T. Yeung

**Affiliations:** Department of Chemistry, University at Albany SUNY, Albany, New York 12222, United States

## Abstract

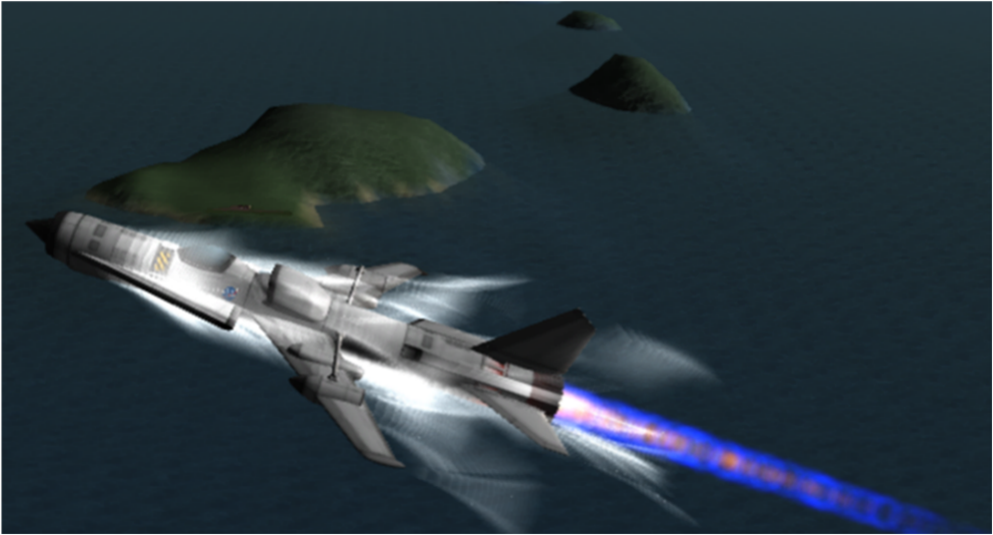

The ideal aeronautical solid-state fuel should possess a high gravimetric heat of combustion (more energy for less weight) and a high volumetric heat of combustion (more room for mission-critical items). In this work, manganese diboride (MnB_2_) demonstrates a high gravimetric heat of combustion of 39.26 kJ/g and the highest volumetric heat of combustion of any known fuel of 208.08 kJ/cm^3^. When compared to the currently used fuel in Space Shuttle rocket boosters and the Space Launch System, aluminum metal, MnB_2_ represents a 26% increase in gravimetric heat of combustion and a 148% increase in volumetric heat of combustion. Surprisingly, the local topology of the inner coordination sphere controls energetic output. A model cluster system analyzed by density functional theory shows that the local environment can contribute to the bulk properties even without physical manifestations in the periodic structure. This high enthalpic performance comes from the metastability of MnB_2_ and demonstrates that transition metals, typically shunned as solid-state fuels, can store potential energy from their high-temperature synthesis through ‘overcoordination’ and violation of their valence shell.

## Introduction

Boron-based fuels have been extensively explored over the last 60+ years, presenting higher enthalpies of combustion over liquid hydrocarbon fuels. These high values stem from the high electropositivity and relatively low weight of elemental boron, which possesses an exceptional volumetric energy density of 136.4 kJ/cm^3^ as compared to normal hydrocarbon-based fuels of 30.7–36.6 kJ/cm^3^.^1−5^ These high volumetric energy densities are especially needed in compact aircraft and rockets to make room for more mission useful equipment. These exceptional values have inspired boron-containing metal compounds as high-density boron-rich fuel sources with the hope that the metals can contribute to the combustion process. In 1985, Gany et al. explored potential boride compounds and found several diborides (metal/boron ratio of MB_2_) with volumetric energy densities above 100 kJ/cm^3^.^2^ AlB_2_-type metal diborides offer the potential scaffold needed to create solid-state energetic materials, which would limit volatility through extended covalent bonding, limit diffusion of moisture in the solid state, and diminish toxicity through absorption. The most notable candidate is MgB_2_ (97.1–103.3 kJ/cm^3^), which has been extensively studied.^6−9^

**Figure 1:**
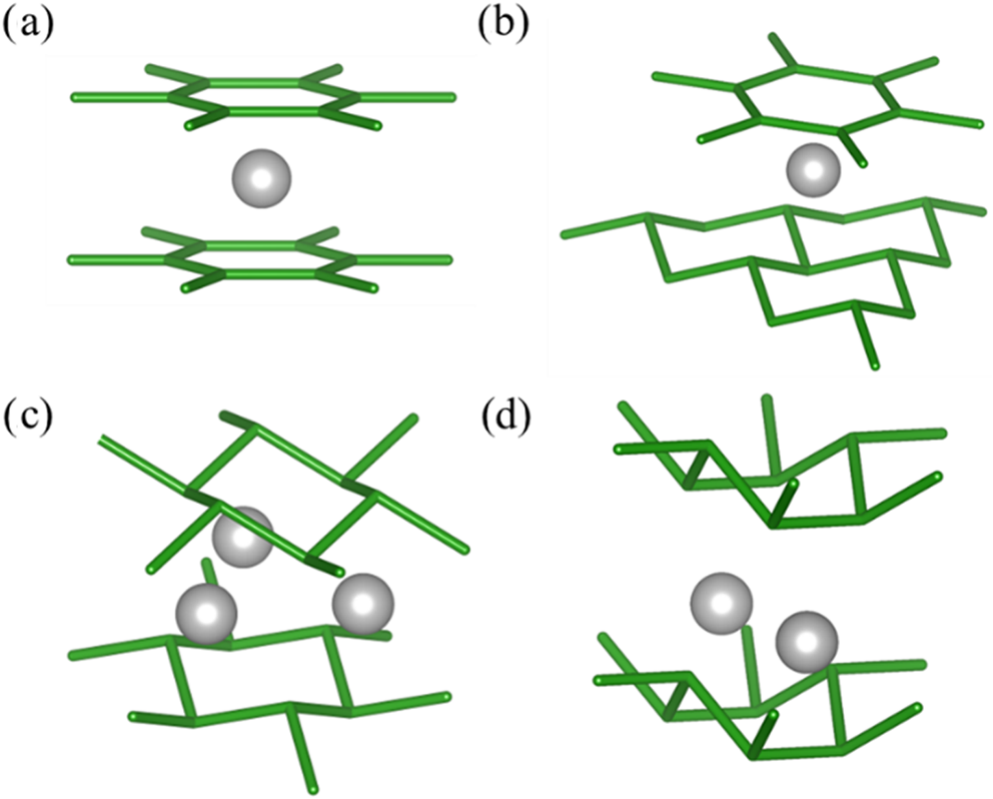
Localized boron surrounding the metal in diborides distorts from the number of d-electrons. (a) AlB_2_-type structures possess planar boron hexagons from little to no d-electrons. (b) Group VI diborides possess alternating planar and chair-confirmation sheets. (c) Group VII diborides possess chair-confirmation sheets. (d) Group VIII diborides possess boat-confirmation sheets.

The crystallization of metal diborides results in several structurally related polymorph (Figure 1), wherein the base hexagonal net of boron is maintained between intermetal layers, but results in different severities of pleating expressed by the boron sheet.^10^ More boron sheet pleating can manifest a reduction in the crystallographic symmetry from hexagonal to orthorhombic crystal systems. The resulting number of metal–boron bonds/coordination number decreases below 12-coordinate with increasing distortion. In the cases of AlB_2_-type diborides such as MgB_2_, AlB_2_, TiB_2_, and TaB_2_, the boron layers remain a flat two-dimensional (2D) sheet and the metal is 12-coordinate. Here, “early” transition metals (with little to no d-electrons) found on the left of the Periodic Table tend to preserve the planar boron structure. Moving to the right toward “late” transition metal diborides begins to perturb the planarity of the boron sheets, resulting in pleating similar to the chair or boat conformations of cyclohexane rings. WB_2_ (Group VI) possesses alternating chair and flat layers, metal is 8-coordinate.^11^ ReB_2_ (Group VII) possesses only chair layers, and the metal is 3-coordinate. Finally, OsB_2_ (Group VIII) results in all the layers of boron distorting into a boat-conformation, metal is 8-coordinate and the overall crystal symmetry descends to orthorhombic instead of hexagonal. Unsurprisingly, no IrB_2_ (Group IV) or any later metal diboride is easily synthesized or stabilized, owing to the extensive distortion.

There are several reasons for this distortion. Burdett et al. argue that it is the interaction between the antibonding orbitals of the transition metal and the p_*z*_ orbital of the borophene sheets that favor this distortion.^12^ As the electron count increases as one moves from early to late transition metals, antibonding orbitals are filled, favoring a distorted structure. Excitingly, they hypothesized that this unfavorable configuration should lead to instability and, thus, a more positive heat of formation. Likewise, Robinson et al. discovered a significant shift in electron density caused by backbonding between Re/Os and the boron–boron π* orbitals which also contributes to a distortion in the boron sheets themselves through lengthened boron–boron bonds.^13^ This uneven distribution of boron–boron bonds favors pleating. A simpler model that complements the two more rigorous aforementioned models is covalent electron counting. The stability of transition metal complexes can be approximated with the 18-electron rule, and rhenium diboride (Group VII) in the flat AlB_2_ polymorph would be 12-coordinate, resulting in a total of 19 electrons. This exceeds the valence shell and the 18-electron rule, and thus, ReB_2_ possesses a distorted chair-configuration boron sheet to lower the coordination number.

This leads to the controversy of manganese diboride, MnB_2_. Manganese is in the same Group VII as rhenium and thus should suffer distortions. Indeed, there has been extensive theory that has predicted that an ReB_2_-type pleated MnB_2_ is the most favorable form at ambient conditions.^14−17^ On the other side, computational modeling argues that an AlB_2_-type planar MnB_2_ is the most favorable form at ambient conditions when factoring in charge–charge interactions. Experimentally, MnB_2_ does not exist at ambient temperature and can only be prepared in the planar AlB_2_-type polymorph at temperatures above 1,100 °C, as seen with the phase diagram. Even then, MnB_2_ is unstable at high temperatures and is difficult to synthesize. In 1959, a short communication by Binder and Post described that the transition from Mn_3_B_4_ to MnB_2_ onset around 1400–1500 °C, but the MnB_2_ product was heavily contaminated with the lower energy and more stable Mn_3_B_4_.^18^ At synthesis temperatures at ambient pressure, MnB_2_ rapidly decomposes to release manganese vapor.^19^ Attaining MnB_2_ has only been achieved using high-pressure high-temperature synthesis or arc melting/rapid heating and cooling to prevent decomposition.^20,21^ Even then, Meng et al. noted that MnB_2_ is so unstable that it will decompose into Mn_3_B_4_ and boron in the middle of high-temperature high-pressure synthesis; the shorter the synthesis, the more MnB_2_ remains.^22^ In terms of thermochemistry, there have been theoretical calculations performed to obtain the enthalpy of formation of MnB_2_ by density functional theory (DFT) which resulted in fairly high values compared to other first-row transition metal diborides.^23^ All previous experimental attempts at thermochemistry for MnB_2_ used impure materials described with weak lines in powder X-ray diffraction (pXRD) and poor crystallinity.^24^

In hindsight, the difficulties in synthesizing MnB_2_ are a reflection of the high instability from the high d-electron count. Following covalent electron counting for the stability of transition metal complexes, the 18-electron rule is violated beyond chromium in a planar AlB_2_-type structure. Manganese (Group VII) coordinated to 12 atoms results in 19 electrons, which exceeds the valence shell and explains why stringent synthetic routes are required to synthesize MnB_2_. Due to the decrease in transition metal complex stability, metastable MnB_2_ has the potential to be used as a solid-state fuel. Here, MnB_2_ wants to crystallize into a distorted structure, but only the high-temperature AlB_2_-type structure is achieved, resulting in metastability. In this paper, we explore the energetic contributions of the delocalized d-electron-induced metastable manganese diboride, MnB_2_.

Here, we introduce the concept of “overcoordination” where a transition metal is forced to accept more coordinative bonds than it prefers through high-temperature synthesis. The discrepancy between the low coordination favored in the local environment and the high coordination favored in the periodic structure stores the high temperatures required to make metastable MnB_2_. In this way, we can translate that high heat of synthesis into a high heat of combustion. We can compare this to the idea of a “Texas” carbon or 5-coordinate carbon, which is a highly metastable state that if somehow stabilized would be an exceptional fuel, or to the intermediate state of the associative mechanisms for ligand exchange where the transition metal is forced to expand its inner coordination number. In other words, MnB_2_ is unstable in the solid state and possesses a high heat of combustion. The energetic profiles of other AlB_2_-type diborides have already been well defined in the work presented by Gany and Netzer, which we use to compare the experimental energy of MgB_2_ and TiB_2_ in this work as a positive control for the combustion matrix.^2^ From our bomb calorimetry measurements, we find that MnB_2_ exhibits an exceptional gravimetric heat of combustion of 39.26 kJ/g and a volumetric heat of combustion of 208.08 kJ/cm^3^. If we compare this to the currently used solid-state fuel of aluminum metal (gravimetric: 31.11 kJ/g; volumetric: 84.00 kJ/cm^3^), which was used in the Space Shuttle’s solid rocket boosters and will be used in the future Space Launch System, we see that MnB_2_ is over 26% higher in gravimetric heat of combustion and is over 148% higher in volumetric heat of combustion.^25−27^ This is, to date, the highest volumetric heat of combustion of any known solid-state fuel discovered.

## Results and Discussion

Given the difficulties in previous attempts to synthesize MnB_2_, we designed a special synthetic procedure to limit manganese evaporation and potential degradation. Indeed, our attempts to prepare MnB_2_ following previous literature attempts in a high-temperature tube furnace at 1250 °C failed (Figure S2).^28^ First, stoichiometric powders of manganese and boron were pressed into pellets and annealed at 1000 °C; this provides an interconnected electrical connection for arc melting and ensures that the resulting pellet will be evenly heated in a rapid manner. Next, the samples were arc melted for no longer than 1 min to limit any potential decomposition, and the copper hearth was kept chilled at 9 °C to rapidly cool the molten ingot and quench the high-temperature AlB_2_-type phase.

**Figure 2:**
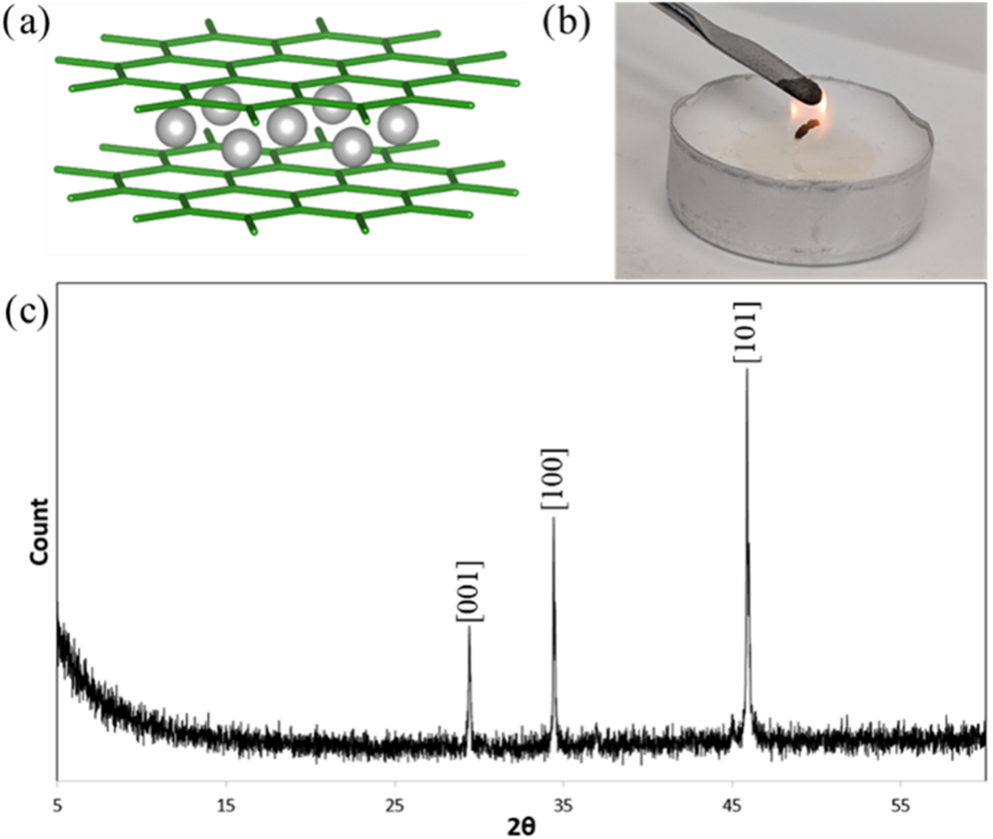
Stable form of MnB_2_ achieved by our synthetic methods. (a) Crystal structure of MnB_2_. (b) Thermal stability of MnB_2_ via exposure to open flame indicates no unwanted combustion without a burning aid. (c) pXRD of phase-pure MnB_2_ synthesized by arc melting.

Powder X-ray diffraction (pXRD) indicates phase-pure MnB_2_ that crystallizes in the *P*6̅ /*mmm* no.191 with lattice parameters *a* = 3.006Å, and *c* = 3.037Å (Figure 2a). The peaks at 29.378, 34.434, and 45.874° 2θ index to [001], [100], and [101], respectively, with no other peaks indicative of the decomposition products such as MnB_4_, MnB, Mn_3_B_4_, or potential oxidation products. The narrow peak width suggests large grains and no nanostructuring that could affect the energetics of this compound. The Mn–B bond distance is a uniform 2.306 Å and the B–B bond distance is a uniform 1.858 Å.^29^ To quantify the distortion, we can measure the length dispersion, as defined by the following equation
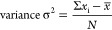
where *x*_i_ is the length, *x̅* is the average of the lengths, and *N* is the number of pairs. If there is a distortion, the bond lengths would vary, and the variance would be positive; if the coordinating environment is isotropic, the variance would be 0. Since both Mn–B and B–B bond distances in the periodic structure are uniform, the variance is exactly 0. The calculated density measured from our X-ray results is 5.3 g/cm^3^, which matches literature results of 5.3 g/cm^3^.^30^ To ensure that our samples are stoichiometric, inductively coupled plasma optical emission spectroscopy was performed and showed an atomic ratio of 32% Mn and 68% B, which is expected for MnB_2_. Because MnB_2_ is a metastable compound, we were concerned about the stability and potential oxidation/hydrolysis in air; its sister compound ReB_2_ is known to degrade in humid atmospheric conditions.^31^ Here, we show that MnB_2_ is stable by examining the Fourier transform infrared spectroscopy (FTIR) of freshly prepared MnB_2_ and comparing it to the FTIR of MnB_2_ that has been aged for >2 weeks in atmospheric conditions. We observe that the fresh sample contains no surface hydroxyls, indicating oxidation and a lack of drift in the fingerprint region. This is maintained after aging, indicating no change in the oxidation of manganese or absorption of atmospheric water (Figure S3). Furthermore, a key component of solid-state fuels is their insensitivity to ensure safe handling, which was tested by bringing some MnB_2_ into an open flame. There was no indication of premature combustion, which confirms that MnB_2_ possesses excellent safety and handleability (Figure 2b).

After synthesizing MnB_2_ and processing into a powder, we performed bomb calorimetry to measure its heat of combustion.^32^ Herein lies a set of challenges: boron and borides (especially refractory borides) do not readily burn in a bomb calorimeter. Incomplete combustion of metal fuels frequently occurs and is attributed to two factors: (1) poor dispersion resulting in fluxing and the agglomeration/segregation of solid fuels^33^ and (2) the presence of a native oxide layer that prevents further combustion. Previous attempts at combusting pure boron required the use of the burning aid paraffin oil (long chain hydrocarbon), which (1) helps disperse the boron/boride and prevents fluxing/melting of powders which can damage the calorimeter and (2) generates both heat and combustion water vapor which has been shown to help penetrate the oxide layer.^4,34,35^ Indeed, modern analysis of solid fuels typically uses a 1:10 ratio of solid fuel with paraffin oil or hydroxyl-terminated polybutadiene as their hydrocarbon burning aid.^36,37^ Building on this work, we wanted to avoid fluxing/incomplete combustion of MnB_2_, and this requires the dispersion of the metal powder in a hydrocarbon-based burning aid. The burning aid of kerosene was chosen due to its high hydrocarbon content, high adiabatic flame temperature, low vapor pressure, low flash point, and its frequent use in jet engine fuel formulations, enabling a more realistic combustion scenario. Targeting 10 wt % ensures that the sample will ignite, and we prepared our dispersions by sonicating MnB_2_ for 2 h and kept the sample agitated to maintain dispersion. Optical microscopy of the MnB_2_ powders in kerosene confirms the role of sonication in dispersing and breaking apart aggregates (Figure S4).

The heat of combustion of kerosene averaged across three replicates was 46.4 kJ/g. The heat of combustion of kerosene can be subtracted from that of our dispersion to yield the true value of MnB_2_. To validate the accuracy of our procedure, MgB_2_ and TiB_2_ were purchased from a commercial vendor and used in calorimeter experiments with comparison to the reported values of 40.21 and 23.85 kJ/g, respectively.^2,38^ The resulting average across three replicates of MgB_2_ was 39.56 ± 0.5 kJ/g (Table S1), indicating complete combustion and results that are within ± 2% of the reported literature value. Three replicates of TiB_2_ produced an average result of 22.85 ± 0.5 kJ/g (Table S2), also indicating complete combustion and corresponding with the reported value within ±4.2% of the reported literature value. With the procedure validated, we prepared our dispersions by sonicating MnB_2_ for 2 h and kept the sample agitated to maintain dispersion. The resulting average across five replicates of MnB_2_ was 39.26 kJ/g (Table 1). Surprisingly, this compares favorably to AlB_2_ (38.08 kJ/g) and MgB_2_ (40.21 kJ/g) despite all of the increased mass from transition metal manganese. Based on the X-ray data, the density was calculated to be 5.3 g/cm^3^, which we can use to convert the gravimetric heat of combustion to a volumetric heat of combustion at an impressive 208.08 kJ/cm^3^.

**Table 1:** Enthalpy of Combustion of 10 wt % MnB_2_ in Kerosene

	trial #1	trial #2	trial #3	trial #4	trial #5	average	standard error
MnB_2_ (g)	0.02567	0.02513	0.02479	0.02511	0.02531	-	-
kerosene (g)	0.22757	0.25638	0.22734	0.23235	0.23099	-	-
percentage MnB_2_ (wt%)	10.14	8.93	9.83	9.75	9.88	-	-
ΔH_c_ MnB_2_ (kJ/g)	36.609	41.534	42.237	37.544	38.379	39.261	0.996
ΔH_c_ MnB_2_ (kJ/cm^3^)	194.032	220.135	223.859	198.984	203.411	208.085	5.277

As described earlier, our product is MnB_2_ in the AlB_2_-type structure with planar boron sheets, resulting in a 12-coordinate environment for manganese. Yet, this should be the unfavorable configuration given the high d-electron count, as the occupied antibonding orbital of MnB_2_ disfavors high coordination. Thus, there is a discrepancy between the local environment and the resulting periodic structure which we call “overcoordination”. To further examine this behavior in detail, we designed an aperiodic cluster model which focuses on the local environment surrounding the Mn. The diborides under investigation were modeled as localized bilayer clusters representing a subregion of the bulk crystal rather than the complete periodic crystal lattice to allow for structural anisotropy. For a given diboride cluster, any deviation between the ideal *P*6̅ /*mmm* symmetry of the crystal structure and that of the cluster represents the stored potential energy.

Here, we started with a hypothetical M_7_(B_24_H_12_)_2_, which can be thought of as a sandwich complex and M = Mg, Ti, or Mn. The boron atoms are arranged analogous to the polyaromatic coronene, and the metals are nestled within each ring. For MnB_2_, TiB_2_, and MgB_2_, the initial structures used for the ab initio density functional theory (DFT) calculations were constructed by extending the unit cell into a 5 × 5 x 2 supercell and subsequently removing all the metal atoms in the bottom layer. Boron atoms around the perimeter of each layer were then removed, resulting in a bilayer structure with M atoms sandwiched between two coronene-like sheets of hexagonal B (hB). The perimeter B atoms in each of the planar sheets were capped with H to maintain the net neutrality of the systems.

Geometry optimizations were performed on diboride systems using the Q-Chem software package with the revised PBE (revPBE) functional and the fit-LANL2DZ effective core potential (ECP), using the generalized gradient approximation (GGA) for the calculation of the exchange correlation energy.^39−45^ The 6–311G* basis set was used for the B and H atoms, while the LANL2DZ basis set was used for the Mg, Ti, and Mn atoms.^45,46^ During each of the geometry optimizations, the B and metal atoms along the perimeter of each sheet were kept fixed, so that only the positions of the local B atoms directly coordinated with the central metal atom were free to change. No such positional restraints were applied to the central metal or perimeter H atoms.

**Figure 3:**
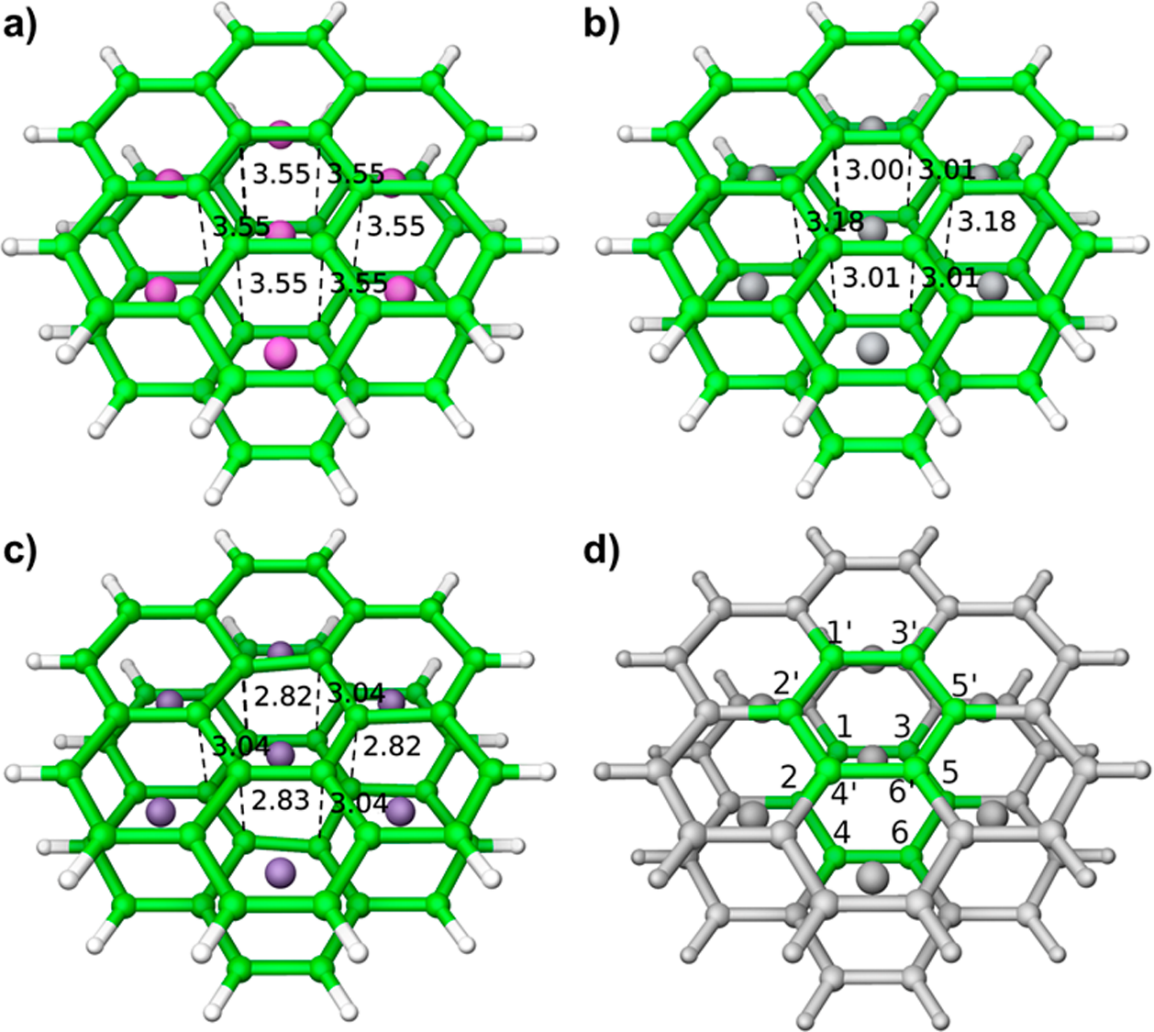
(a) MgB_2_ local cluster model showing coordination with Group II valence electrons. The inherent lack of d-electrons around magnesium is reflected in the uniform interatomic distance between the boron sheets. (b) TiB_2_ local cluster model showing coordination (16 valence electrons) with Group IV d-electrons causes a very subtle boat-like deformation, represented by the shortened interatomic distances of boron atoms of B1 and B4. (c) MnB_2_ local cluster model showing overcoordination (19 valence electrons) with Group VII d-electrons causes the observed chair deformation, represented by the shortened interatomic distances of boron atoms of B1, B3, and B5. (d) Local cluster legend showing the positions of assigned boron atoms around the center.

Upon relaxation of the Mg_7_(B_24_H_12_)_2_, the resulting structure (Figure 3a) is planar with Mg–B bond distances of 2.49Å and B–B bond distances of 1.75Å, and this compares favorably to the crystallographic Mg–B bond distances of 2.50Å and B–B bond distances of 1.78Å. There is no perturbation in the distance between boron layers in the B1–B1′, B2–B2′, B3–B3′, B5–B5′, and B6–B6′ resulting in a variance of 0 (Table 2). This indicates that the planar configuration found in periodic MgB_2_ matches the desired local configuration. Repeating the computational treatment to Ti_7_(B_24_H_12_)_2_ resulted in only minimal deformation of two boron atoms (Figure 3b), with Ti–B bond distances of [2.32, 2.17, 2.32, 2.18, 2.32, 2.18]Å and uniform B–B bond distances of 1.71Å, compared to the crystallographic Ti–B bond distance of 2.38Å and B–B bond distance of 1.75Å. A slight perturbation in the distance between boron layers in the B2–B2′, B3–B3′, B5–B5′, and B6–B6′ bonds show a shift inward toward the metal center resulting in an interatomic sheet distance of 3.01Å. The B–B’ variance is subtle 0.00795 Å^2^, which indicates a subtle distortion. As TiB_2_ has more d-electrons than MgB_2_, the local environment favors some distortion but is overall planar.

**Table 2:** B–B’ Interlayer Distances

	distance (Å)		
B atoms	MgB_2_	TiB_2_	MnB_2_
1–1′	3.55	3.18	2.82
2–2′	3.55	3.01	3.04
3–3′	3.55	3.01	3.04
4–4′	3.55	3.01	2.82
5–5′	3.55	3.00	2.83
6–6′	3.55	3.18	3.04
variance	0	0.007	0.014

On the other hand, upon relaxation of the Mn_7_(B_24_H_12_)_2_, the resulting interior B_6_ ring is not planar with Mn–B bond distances of [2.28, 2.28, 2.34, 2.28, 2.28, 2.34, 2.28, 2.28, 2.34, 2.28, 2.28, and 2.34]Å, and B–B bond distances of [1.72, 1.72, 1.71, 1.72, 1.72, 1.71, 1.72, 1.72, 1.71, 1.72, 1.72, and 1.71]Å. This compares unfavorably to the crystallographic Mn–B bond distances of 2.31Å and B–B bond distances of 1.73Å. A major perturbation in the distance between boron layers in the B1–B1′, B4–B4′, and B5–B5′ results in a variance of 0.014 Å^2^, nearly double that of TiB_2_. These two variances in both the Mn–B and B–B bond lengths result in the adoption of a deformed chair conformation. (Figure 3c) Expectedly, the high d-electron count manifests in a distortion from planarity. The observed deformations are normally not seen in the periodic simulation because the other metal atoms would prevent the movement and rearrangement of the upper and lower boron atoms. In the local environment, we get a true sense of the forces being exerted on the boron atoms with the addition of excess d-electrons. The complete list of interatomic distances between the B atoms in the central top and bottom layers of the diborides is presented in Table 2.

**Figure 4:**
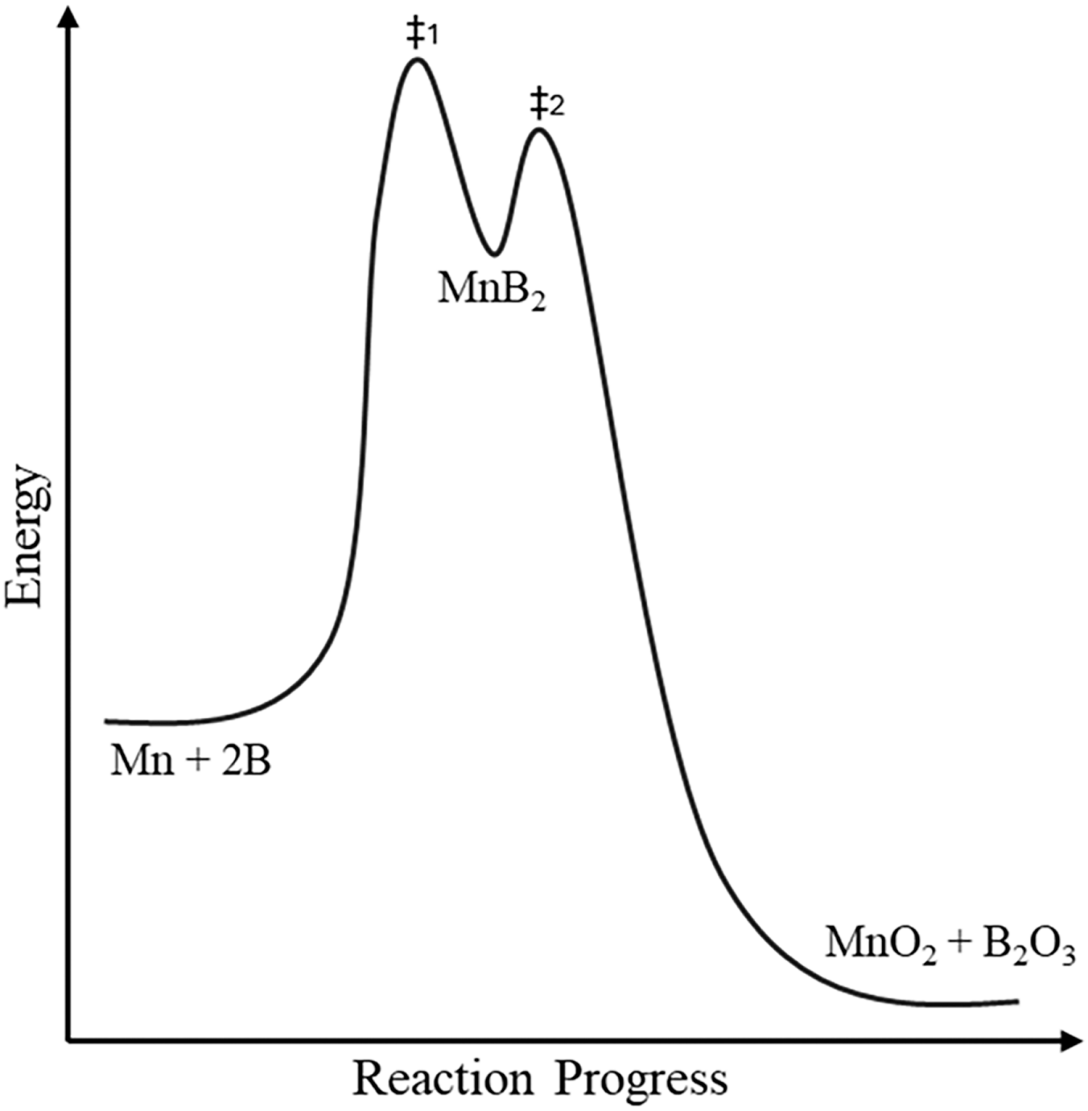
Example reaction coordination diagram emphasizing the intermediate metastable state of overcoordinated MnB_2_.

Putting this all together, we hypothesize that the exceptionally high heat of combustion of MnB_2_ comes from this discrepancy between the preferred coordination of local Mn in MnB_2_ versus the periodic coordination that is experimentally synthesizable. This is the concept of “overcoordination” where periodic MnB_2_ coordination of 12 exceeds the favored local coordination of 6. This would be the energetic equivalent of local minima. This is similar in concept to the reaction coordinate of an associative mechanism for transition metal complexes. In the associative mechanism, the intermediate state is overcoordinated with both the entering and leaving group coordinated to the transition metal. Visually, we can represent the metastable intermediate state as a higher-energy reaction product on a reaction coordinate diagram, depicting the formation and oxidative combustion of MnB_2_ (Figure 4), whereby rapidly cooling the molten elements while the atoms are still in a state of flux will force them to assume the most ordered position available before being frozen into place. Here, the high heat from the burning aid overcomes the energy barrier of MnB_2_ to form oxides. Moreover, this demonstrates that transition metals can be used as fuel atoms in the solid state beyond clusters.^47^

## Conclusions

**Figure 5:**
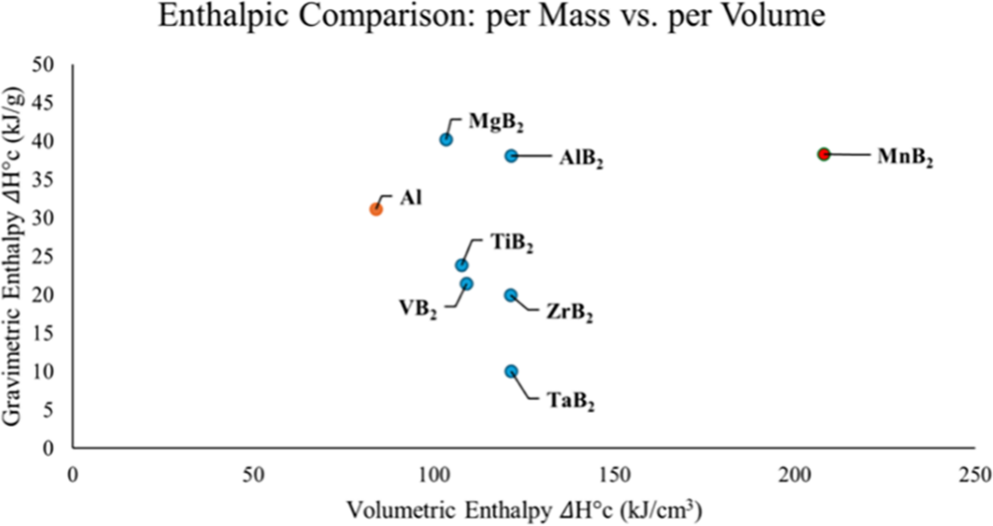
Enthalpic comparison of transition metal borides by mass and volume. The orange data point represents aluminum currently used in rocketry. Blue data points represent transition metal diborides previously investigated^2,38^ The red data point represents the experimentally verified metastable MnB_2_.

Beyond the energetic reach of elemental boron lies a field of energetically rich metastable boron compounds that are yet to be explored. Manganese diboride’s overcoordination outside the 18-electron rule suggests that transition metals can achieve higher-energy-density fuels. Excess d-electrons play a key role in raising the potential energy of diborides and will dictate the overcoordination felt across the planar hexagonal boron sheets. Here, we have demonstrated that we can take the heat of synthesis required for metastable MnB_2_ and apply it directly to the heat of combustion. In turn, MnB_2_ possesses the highest volumetric heat of combustion of all known fuels, which can be compared with an energy density plot (Figure 5).

## Supporting Information Available

Experimental methods; enthalpy of combustion of 10 wt % MgB_2_ in kerosene; enthalpy of combustion of 10 wt % TiB_2_ in kerosene; MB_2_ localized metal cluster with labeled boron atoms; MnB_2_ high-temperature furnace synthesis in aluminum flux, and supporting references (PDF)

## References

[ref1] Ao W.; Wang Y. Effect of Gas Generator Pressure on the Physicochemical, Oxidation and Combustion Characteristics of Boron-Based Propellant Primary Combustion Products. J. Therm. Anal. Calorim. 2017, 129 (3), 1865–1874. 10.1007/s10973-017-6339-8.

[ref2] Gany A.; Netzer D. W. Fuel Performance Evaluation for the Solid-Fueled Ramjet. Int. J. Turbo Jet Engines 1985, 2 (2), 661–668. 10.1515/TJJ.1985.2.2.157.

[ref3] Zhang X.; Pan L.; Wang L.; Zou J.-J. Review on Synthesis and Properties of High-Energy-Density Liquid Fuels: Hydrocarbons, Nanofluids and Energetic Ionic Liquids. Chem. Eng. Sci. 2018, 180, 95–125. 10.1016/j.ces.2017.11.044.

[ref4] Eggersgluess W.; Monroe A. G.; Parker W. G. The Heat of Formation of Boron Trioxide. Trans. Faraday Soc. 1949, 45, 661–668. 10.1039/tf9494500661.

[ref5] Rossini F. D. Heats-of-Combustion and of Formation of the Normal Paraffin Hydrocarbons in the Gaseous State, and the Energies of Their Atomic Linkages. J. Res. Natl. Bur. Stand. 1934, 13 (1), 21–35. 10.6028/jres.013.004.

[ref6] Liang D.; Xiao R.; Li H.; Liu J. Heterogeneous Decomposition and Oxidation during Combustion of Magnesium Diboride Particles. Acta Astronaut 2018, 153, 159–165. 10.1016/j.actaastro.2018.10.026.

[ref7] Guo Y.; Zhang W.; Zhou X.; Bao T. Magnesium Boride Sintered as High-Energy Fuel. J. Therm. Anal. Calorim. 2013, 113 (2), 787–791. 10.1007/s10973-012-2832-2.

[ref8] Pal Y.; Palateerdham S. K.; Mahottamananda S. N.; Sivakumar S.; Ingenito A. Combustion Performance of Hybrid Rocket Fuels Loaded with MgB2 and Carbon Black Additives. Propul. Power Res. 2023, 12 (2), 212–226. 10.1016/j.jppr.2022.11.003.

[ref9] Air-Augmented Combustion of Boron and Boron-Metal Alloy. https://apps.dtic.mil/sti/citations/AD0886550 (accessed February 17, 2025).

[ref10] Albert B.; Hillebrecht H. Boron: Elementary Challenge for Experimenters and Theoreticians. Angew. Chem., Int. Ed. 2009, 48 (46), 8640–8668. 10.1002/anie.200903246. 19830749

[ref11] Frotscher M.; Klein W.; Bauer J.; Fang C.-M.; Halet J.-F.; Senyshyn A.; Baehtz C.; Albert B. M2B5 or M2B4? A Reinvestigation of the Mo/B and W/B System. Z. Anorg. Allg. Chem. 2007, 633 (15), 2626–2630. 10.1002/zaac.200700376.

[ref12] Burdett J. K.; Canadell E.; Miller G. J. Electronic Structure of Transition-Metal Borides with the AlB2 Structure. J. Am. Chem. Soc. 1986, 108 (21), 6561–6568. 10.1021/ja00281a020.

[ref13] Robinson P. J.; Liu G.; Ciborowski S.; Martinez-Martinez C.; Chamorro J. R.; Zhang X.; McQueen T. M.; Bowen K. H.; Alexandrova A. N. Mystery of Three Borides: Differential Metal–Boron Bonding Governing Superhard Structures. Chem. Mater. 2017, 29 (23), 9892–9896. 10.1021/acs.chemmater.7b04378.

[ref14] Wang X. F.; Wang Y. X.; Wu H.; Xie W. N.; Zhang Y. X.; Wang Z. Theoretical Prediction of Phase Transition, Mechanical and Electronic Properties of Manganese Diboride under Pressure. Appl. Phys. A 2024, 130 (6), 414 10.1007/s00339-024-07558-9.

[ref15] Aydin S.; Simsek M. First-Principles Calculations of MnB2, TcB2, and ReB2 within the ReB2-Type Structure. Phys. Rev. B 2009, 80 (13), 134107 10.1103/PhysRevB.80.134107.

[ref16] Wang B.; Li X.; Wang Y. X.; Tu Y. F. Phase Stability and Physical Properties of Manganese Borides: A First-Principles Study. J. Phys. Chem. C 2011, 115 (43), 21429–21435. 10.1021/jp2073683.

[ref17] Fan J.; Bao K.; Jin X.; Meng X.; Duan D.; Liu B.; Cui T. How to Get Superhard MnB2: A First-Principles Study. J. Mater. Chem. 2012, 22 (34), 17630–17635. 10.1039/c2jm31385e.

[ref18] Binder I.; Post B. Manganese Diboride. Acta Crystallogr. 1960, 13 (4), 356 10.1107/S0365110X60000820.

[ref19] Lönnberg B. Thermal Expansion Studies on the Group IV–VII Transition Metal Diborides. J. Less Common Met. 1988, 141 (1), 145–156. 10.1016/0022-5088(88)90219-6.

[ref20] Cadeville M. C. Proprietes Magnetiques Des Diborures de Manganese et de Chrome: MnB2 et CrB2. J. Phys. Chem. Solids 1966, 27 (4), 667–670. 10.1016/0022-3697(66)90217-4.

[ref21] Gou H.; Steinle-Neumann G.; Bykova E.; Nakajima Y.; Miyajima N.; Li Y.; Ovsyannikov S. V.; Dubrovinsky L. S.; Dubrovinskaia N. Stability of MnB2 with AlB2-Type Structure Revealed by First-Principles Calculations and Experiments. Appl. Phys. Lett. 2013, 102 (6), 061906 10.1063/1.4792273.

[ref22] Meng X.; Bao K.; Zhu P.; He Z.; Tao Q.; Li J.; Mao Z.; Cui T. Manganese Borides Synthesized at High Pressure and High Temperature. J. Appl. Phys. 2012, 111 (11), 112616 10.1063/1.4726230.

[ref23] Colinet C.; Tedenac J.-C. Enthalpies of Formation of Transition Metal Diborides: A First Principles Study. Crystals 2015, 5 (4), 562–582. 10.3390/cryst5040562.

[ref24] Kleppa O. J.; Sato S. New Applications of High-Temperature Solution Calorimetry III. Enthalpies of Formation of Mn2B, MnB, and MnB2. J. Chem. Thermodyn. 1982, 14 (2), 133–143. 10.1016/0021-9614(82)90025-8.

[ref25] Sundaram D. S.; Puri P.; Yang V. A General Theory of Ignition and Combustion of Nano- and Micron-Sized Aluminum Particles. Combust. Flame 2016, 169, 94–109. 10.1016/j.combustflame.2016.04.005.

[ref26] Creech S.; Stough R. W.; Hitt D. In NASA’s Space Launch System for High-C3 Science Missions, AIAA Propulsion and Energy 2020 Forum; American Institute of Aeronautics and Astronautics, 2020.

[ref27] Thomason H. In Space Shuttle System Solid Rocket Booster, Joint Space Mission Planning and Execution Meeting; American Institute of Aeronautics and Astronautics, 1973.

[ref28] Higashi I.; Takahashi Y.; Atoda T. Crystal Growth of Borides and Carbides of Transition Metals from Molten Aluminum Solutions. J. Cryst. Growth 1976, 33 (2), 207–211. 10.1016/0022-0248(76)90044-0.

[ref29] Momma K.; Izumi F. VESTA 3 for Three-Dimensional Visualization of Crystal, Volumetric and Morphology Data. J. Appl. Crystallogr. 2011, 44 (6), 1272–1276. 10.1107/S0021889811038970.

[ref30] None Available. Materials Data on MnB2 by Materials Project, 2020 10.17188/1277089.

[ref31] Orlovskaya N.; Xie Z.; Klimov M.; Heinrich H.; Restrepo D.; Blair R.; Suryanarayana C. Mechanochemical Synthesis of ReB2 Powder. J. Mater. Res. 2011, 26 (21), 2772–2779. 10.1557/jmr.2011.249.

[ref32] 6725 Semimicro Calorimeter. Parr Instrument Company. https://www.parrinst.com/products/oxygen-bomb-calorimeters/6725-semimicro-calorimeter-2/ (accessed May 21, 2025).

[ref33] Tran Q.; Altman I.; Dube P.; Malkoun M.; Sadangi R.; Koch R.; Pantoya M. L. Direct Demonstration of Complete Combustion of Gas-Suspended Powder Metal Fuel Using Bomb Calorimetry. Meas. Sci. Technol. 2022, 33 (4), 047002 10.1088/1361-6501/ac47bc.

[ref34] Xi J.; Liu J.; Wang Y.; Liang D.; Li H.; Zhou J. Role of Oxalic Acid in Promoting Ignition and Combustion of Boron: An Experimental and Theoretical Study. Propellants, Explos., Pyrotech. 2014, 39 (6), 844–851. 10.1002/prep.201400048.

[ref35] Young G.; Roberts C. W.; Stoltz C. A. Ignition and Combustion Enhancement of Boron with Polytetrafluoroethylene. J. Propul. Power 2015, 31 (1), 386–392. 10.2514/1.B35390.

[ref36] Epshteyn A.; Miller J. B.; Pettigrew K. A.; Stroud R. M.; Purdy A. P. Surface Passivated Air and Moisture Stable Mixed Zirconium Aluminum Metal-Hydride Nanoparticles. MRS Proc. 2007, 1056, 316 10.1557/PROC-1056-HH03-16.

[ref37] Finn M. T.; Chaloux B. L.; Epshteyn A. Exploring the Effects of Reaction Conditions on Morphology and Stability of Sonochemically Generated Ti–Al–B Fuel Powders. Energy Fuels 2020, 34 (9), 11373–11380. 10.1021/acs.energyfuels.0c01050.

[ref38] Bondarchuk S. S.; Matveev A. E.; Promakhov V. V.; Vorozhtsov A. B.; Zhukov A. S.; Zhukov I. A.; Ziatdinov M. H. Synthesis and Properties of Energetics Metal Borides for Hybrid Solid-Propellant Rocket Engines. In Proceedings of the Scientific-Practical Conference “Research and Development - 2016”; Anisimov K. V.; Dub A. V.; Kolpakov S. K.; Lisitsa A. V.; Petrov A. N.; Polukarov V. P.; Popel O. S.; Vinokurov V. A., Eds.; Springer International Publishing: Cham, 2018; pp 511–519.

[ref39] Epifanovsky E.; Gilbert A. T. B.; Feng X.; Lee J.; Mao Y.; Mardirossian N.; Pokhilko P.; White A. F.; Coons M. P.; Dempwolff A. L.; Gan Z.; Hait D.; Horn P. R.; Jacobson L. D.; Kaliman I.; Kussmann J.; Lange A. W.; Lao K. U.; Levine D. S.; Liu J.; McKenzie S. C.; Morrison A. F.; Nanda K. D.; Plasser F.; Rehn D. R.; Vidal M. L.; You Z.-Q.; Zhu Y.; Alam B.; Albrecht B. J.; Aldossary A.; Alguire E.; Andersen J. H.; Athavale V.; Barton D.; Begam K.; Behn A.; Bellonzi N.; Bernard Y. A.; Berquist E. J.; Burton H. G. A.; Carreras A.; Carter-Fenk K.; Chakraborty R.; Chien A. D.; Closser K. D.; Cofer-Shabica V.; Dasgupta S.; de Wergifosse M.; Deng J.; Diedenhofen M.; Do H.; Ehlert S.; Fang P.-T.; Fatehi S.; Feng Q.; Friedhoff T.; Gayvert J.; Ge Q.; Gidofalvi G.; Goldey M.; Gomes J.; Gonzalez-Espinoza C. E.; Gulania S.; Gunina A. O.; Hanson-Heine M. W. D.; Harbach P. H. P.; Hauser A.; Herbst M. F.; Hernandez Vera M.; Hodecker M.; Holden Z. C.; Houck S.; Huang X.; Hui K.; Huynh B. C.; Ivanov M.; Jasz A.; Ji H.; Jiang H.; Kaduk B.; Kahler S.; Khistyaev K.; Kim J.; Kis G.; Klunzinger P.; Koczor-Benda Z.; Koh J. H.; Kosenkov D.; Koulias L.; Kowalczyk T.; Krauter C. M.; Kue K.; Kunitsa A.; Kus T.; Ladjanszki I.; Landau A.; Lawler K. V.; Lefrancois D.; Lehtola S.; Li R. R.; Li Y.-P.; Liang J.; Liebenthal M.; Lin H.-H.; Lin Y.-S.; Liu F.; Liu K.-Y.; Loipersberger M.; Luenser A.; Manjanath A.; Manohar P.; Mansoor E.; Manzer S. F.; Mao S.-P.; Marenich A. V.; Markovich T.; Mason S.; Maurer S. A.; McLaughlin P. F.; Menger M. F. S. J.; Mewes J.-M.; Mewes S. A.; Morgante P.; Mullinax J. W.; Oosterbaan K. J.; Paran G.; Paul A. C.; Paul S. K.; Pavosevic F.; Pei Z.; Prager S.; Proynov E. I.; Rak A.; Ramos-Cordoba E.; Rana B.; Rask A. E.; Rettig A.; Richard R. M.; Rob F.; Rossomme E.; Scheele T.; Scheurer M.; Schneider M.; Sergueev N.; Sharada S. M.; Skomorowski W.; Small D. W.; Stein C. J.; Su Y.-C.; Sundstrom E. J.; Tao Z.; Thirman J.; Tornai G. J.; Tsuchimochi T.; Tubman N. M.; Veccham S. P.; Vydrov O.; Wenzel J.; Witte J.; Yamada A.; Yao K.; Yeganeh S.; Yost S. R.; Zech A.; Zhang I. Y.; Zhang X.; Zhang Y.; Zuev D.; Aspuru-Guzik A.; Bell A. T.; Besley N. A.; Bravaya K. B.; Brooks B. R.; Casanova D.; Chai J.-D.; Coriani S.; Cramer C. J.; Cserey G.; DePrince A. E.; DiStasio R. A.; Dreuw A.; Dunietz B. D.; Furlani T. R.; Goddard W. A.; Hammes-Schiffer S.; Head-Gordon T.; Hehre W. J.; Hsu C.-P.; Jagau T.-C.; Jung Y.; Klamt A.; Kong J.; Lambrecht D. S.; Liang W.; Mayhall N. J.; McCurdy C. W.; Neaton J. B.; Ochsenfeld C.; Parkhill J. A.; Peverati R.; Rassolov V. A.; Shao Y.; Slipchenko L. V.; Stauch T.; Steele R. P.; Subotnik J. E.; Thom A. J. W.; Tkatchenko A.; Truhlar D. G.; Van Voorhis T.; Wesolowski T. A.; Whaley K. B.; Woodcock H. L.; Zimmerman P. M.; Faraji S.; Gill P. M. W.; Head-Gordon M.; Herbert J. M.; Krylov A. I. Frontiers of Quantum Chemistry: An Overview of Developments in the Q-Chem 5 Package. Journal of Chemical Physics 2021, 155, 084801 10.1063/5.0055522. 34470363 PMC9984241

[ref40] Hay P. J.; Martin R. L. Theoretical Studies of the Structures and Vibrational Frequencies of Actinide Compounds Using Relativistic Effective Core Potentials with Hartree–Fock and Density Functional Methods: UF6, NpF6, and PuF6. J. Chem. Phys. 1998, 109 (10), 3875–3881. 10.1063/1.476988.

[ref41] Hay P. J.; Wadt W. R. Ab Initio Effective Core Potentials for Molecular Calculations. Potentials for the Transition Metal Atoms Sc to Hg. J. Chem. Phys. 1985, 82 (1), 270–283. 10.1063/1.448799.

[ref42] Hay P. J.; Wadt W. R. Ab Initio Effective Core Potentials for Molecular Calculations. Potentials for K to Au Including the Outermost Core Orbitals. J. Chem. Phys. 1985, 82 (1), 299–310. 10.1063/1.448975.

[ref43] Hay P. J. Ab Initio Studies of Excited States of Polyatomic Molecules Including Spin-orbit and Multiplet Effects: The Electronic States of UF6. J. Chem. Phys. 1983, 79 (11), 5469–5482. 10.1063/1.445665.

[ref44] Wadt W. R.; Hay P. J. Ab Initio Effective Core Potentials for Molecular Calculations. Potentials for Main Group Elements Na to Bi. J. Chem. Phys. 1985, 82 (1), 284–298. 10.1063/1.448800.

[ref45] Zhang Y.; Yang W. Comment on “Generalized Gradient Approximation Made Simple”. Phys. Rev. Lett. 1998, 80 (4), 890 10.1103/PhysRevLett.80.890.

[ref46] Binkley J. S.; Pople J. A.; Hehre W. J. Self-Consistent Molecular Orbital Methods. 21. Small Split-Valence Basis Sets for First-Row Elements. J. Am. Chem. Soc. 1980, 102 (3), 939–947. 10.1021/ja00523a008.

[ref47] Bacon A. M.; Tomlinson W.; Hooper J. P.; Zdilla M. J. Titanium(II) as a Fuel Atom in Energetic Materials. Inorg. Chem. 2023, 62 (24), 9285–9290. 10.1021/acs.inorgchem.2c04367. 37267586

