# Supplementary material for: Violations of Coordination: Exploring Metastable Diborides via Energetic Transition Metals
Source: J Am Chem Soc. 2025 May 2;147(19):16578–84. doi: 10.1021/jacs.5c04066 (PMC12082694; doi:10.1021/jacs.5c04066)
Supplement: Supplementary file 1 — ja5c04066_si_001.pdf [file ja5c04066_si_001.pdf]

Supplemental Information

Violations of Coordination: Exploring Metastable Diborides via Energetic Transition Metals

Joseph T. Doane[1], Gregory M. John[1], Alma Kolakji[1], Abraham A. Rosenberg[1], Yiren Zhang[1]; Alan A. Chen[1], and Michael T. Yeung[1*]

[1] Department of Chemistry; University at Albany SUNY; Albany, New York, 12222

[*] Corresponding authors: mtyeung@albany.edu

Experimental Methods

Manganese diboride synthesis

A molar ratio of 1:2 manganese (Strem Chemicals, Inc.; 99+% purity) to boron (Alfa Aesar; 325 mesh; 98% purity) powder is weighed out and ground together in an agate mortar and pestle. Approximately 0.3 grams of mixed powders are loaded into a steel die set and compressed under 5000 psi with a hydraulic press. The pellet is carefully extracted from the die press into an alumina crucible and heated in a tube furnace under argon. The heating profile is as follows: hold at 25°C for 6 hours, ramp to 1000°C at 5°C/min, hold at 1000°C for 24 hours, cool at a rate of 5°C/min until 25°C. The pellet was then removed from the furnace and loaded into an MAM-1 arc melter. A zirconium ingot was used as an oxygen getter, which is heated before melting the pellet into an ingot at >2000°C. The pellet heats to molten over 10-20 seconds and cools back to room temperature over 10-20 seconds due to the cooling of the copper plate with a chiller set to 9°C. The MnB_2 ingot is then crushed in a high impact mortar and ground further into a powder in a sapphire mortar and pestle. pXRD (**Figure 2**) is used to confirm the crystal structure to be AlB_2-type hexagonal in the space group P6/mmm no. 191.

Bomb Calorimetry

Samples for calorimetry were prepared using kerosene as a burning aid to provide enough thermal energy to begin the ignition of the metal powders. Ignition of neat MnB_2 metal powder pellets does not occur owing to their refractory nature. On the other hand, ignition of neat MgB_2 metal powder results in fluxing heat release that melts the sample holder, sample loop, and wire into a solid ingot. Approximately 0.025g of boride powder [MnB_2, TiB_2 (Sigma Aldrich; 99+%

purity), or MgB_2 (Beantown Chemical; 99% purity)] is combined with approximately 0.225g of kerosene into a 2 mL centrifuge tube. The tube is sealed using parafilm and put into the sonicator bath for a minimum of 2 hours; maximum of 4 hours. Upon removal from the sonicator bath, the sample is finely dispersed in the kerosene but will settle over time so it is constantly agitated. The sample is then mixed via pipette action before two drops, ranging from 0.30-0.55g combined mass, are loaded into the sample cup. An ignition wire is weighed, typically ranging between 0.015-0.017g, and attached to the calorimeter leads as per the user manuals description. Three purge cycles are performed on the closed vessel using 30 psi of pure oxygen, to ensure any nitrogen in the vessel has been removed that could cause the formation of nitric acid. The calorimeter is charged to 30 psi of oxygen and fully sealed before attaching the ignition wires and submerging in 450 mL of DI water. A Parr 6725 Semimicro Calorimeter runs the ignition cycle and automatically tracks temperature changes over time.[46] The resulting total change in water temperature is used to calculate the enthalpy of combustion of the boride powders (**Equation S1**).

Equation S1: Enthalpy of Combustion of Diborides in Kerosene

$$H_c(MB_2) = ((\Delta T \cdot Cp) - (\Delta m_w \cdot 1400\ \text{cal/g}) - (\delta m_k * H_c(k)) / \delta m_B$$

Where;
$H_c(MB_2)$ = Enthalpy of combustion of metal diboride
ΔT = Change in water temperature
Cp = Heat Capacity of the calorimeter (standardized to benzoic acid)
Δm_w = Change in Mass of Wire Post Burning
δm_k = Partition of solution mass attributed to kerosene
$H_c(k)$ = Average enthalpy of combustion of kerosene found experimentally
δm_B = Partition of solution mass attributed to metal diboride

Inductively Coupled Plasma – Atomic Emission Spectroscopy

Elemental analysis for the ratio of manganese to boron was performed by dissolving approximately 5 mg of MnB_2 in 10 mL of 2% aqueous nitric acid solution for a maximum of 24 hours. Once the solution is homogenous, 1 mL of sample is further diluted in 9 mL of deionized water to for the 100x dilution sample that will be sampled into the ICP-AES.

Sample Characterization Instrumentation

Sample characterization was performed using powder X-ray diffraction (Rigaku Miniflex 6T), Ionization Coupled Plasma – Atomic Emission Spectroscopy (Shimadzu ICPE 9800 Series), Oxygen Bomb Calorimetry (Parr 6725 Semimicro Calorimeter), Zeiss Vert Optical Microscope, and Fourier Transform Infrared Spectrophotometer (Shimadzu IRTracer-100).

Theoretical Methods

Ab initio density functional theory (DFT) calculations were performed with the generalized gradient approximation (GGA) optimize the geometries of bi-layer clusters of MB_2 (M=Mg, Ti, Mn) using the Q-Chem[1] software package with the revised PBE (revPBE[2]) functional and the fit-LANL2DZ[3-7] effective core potential (ECP). The 6-311G*[8] basis set was used for the B and H atoms, while the LANL2DZ[7] basis set was used for the Mg, Ti, and Mn atoms.

The initial atomic coordinates for each cluster were constructed by extending the unit cell into a 5x5x2 supercell and subsequently removing all but the central-most M atom in the top layer and all the M atoms in the bottom layer. Boron atoms around the perimeter of each layer

were then removed, resulting in a bi-layer structure with M atoms sandwiched between two sheets of hexagonal B (hB). The perimeter B atoms in each of the planar sheets were capped with H to maintain net neutrality of the systems.

During each of the geometry optimizations, only the positions of the 12 B atoms directly coordinated with the central M atom (as well as the central M atom itself) were free to change, while the positions of all the other atoms were kept fixed.

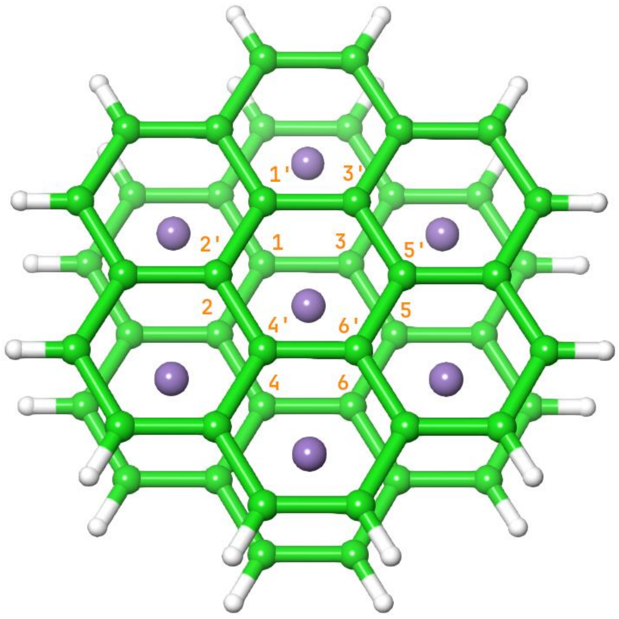

Figure S1: MB_2 localized metal cluster with labelled boron atoms.

Tables and Figures:

Table S1: Enthalpy of Combustion of 10 wt% MgB_2 in Kerosene

	Trial #1	Trial #2	Trial #3	Average	Standard Error
**MgB_2 (g)**	0.02309	0.0251	0.02554	-	-
**Kerosene (g)**	0.23039	0.23289	0.22962	-	-
**Percentage MgB_2 (wt%)**	9.11	9.73	10.01	-	-
**ΔH_c MgB2 (kJ/g)**	38.664	38.767	41.269	39.567	0.852
**ΔH_c MgB2 (kJ/cm3)**	99.365	99.632	106.060	101.686	2.189

Table S2: Enthalpy of Combustion of 10 wt% TiB_2 in Kerosene

	Trial #1	Trial #2	Trial #3	Average	Standard Error
**TiB_2 (g)**	0.02513	0.02474	0.02535	--	--
**Kerosene (g)**	0.22851	0.22930	0.22946	--	--
**Percentage TiB_2 (wt%)**	9.91	9.74	9.95	--	--
**ΔH_c TiB_2 (kJ/g)**	21.448	21.843	25.278	22.857	1.216
**ΔH_c TiB_2 (kJ/cm3)**	96.947	98.730	114.259	103.312	5.498

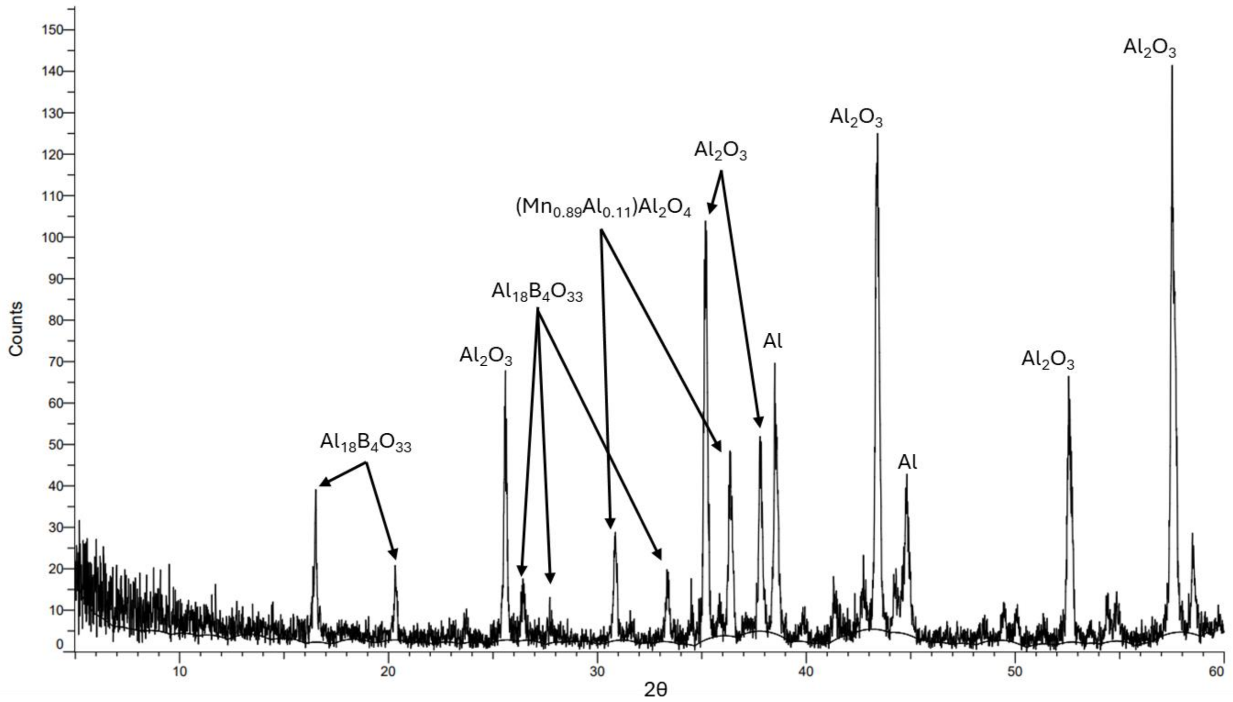

Figure S2: MnB_2 High Temperature Furnace Synthesis in aluminum Flux at 1250°C for 12 hours, following literature methods.[27] MnB_2 does not form below 1400-1500°C.[18] Use of aluminum flux causes formation of side products and high aluminum oxide impurity that originates from the alumina crucible that the product fused to during the heating process.

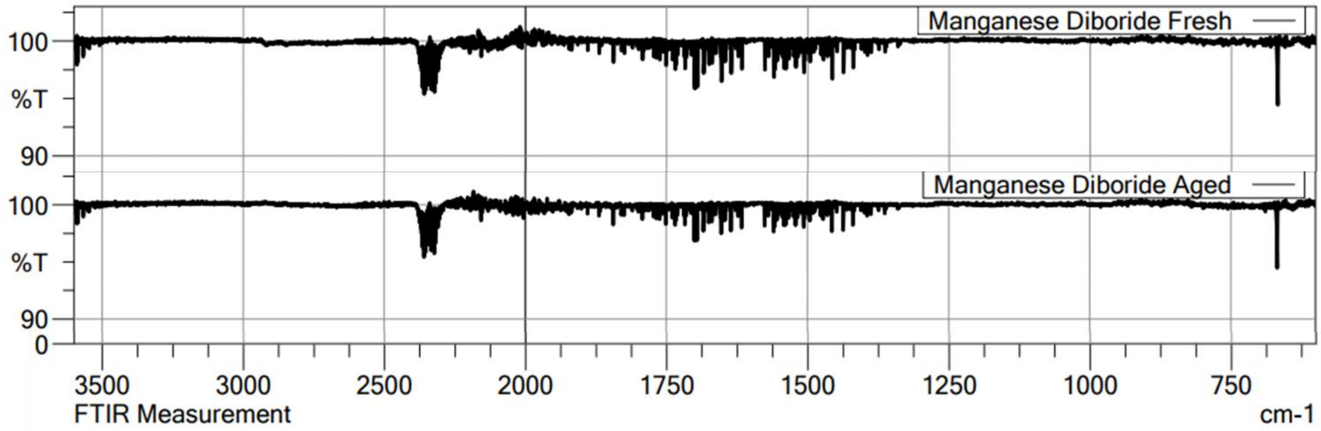

Figure S3: FTIR scan of MnB_2 freshly ground from arc melted ingot and then aged for >2 weeks in atmospheric conditions. No detection of hydroxyl peaks, material degradation, or water contamination are present.

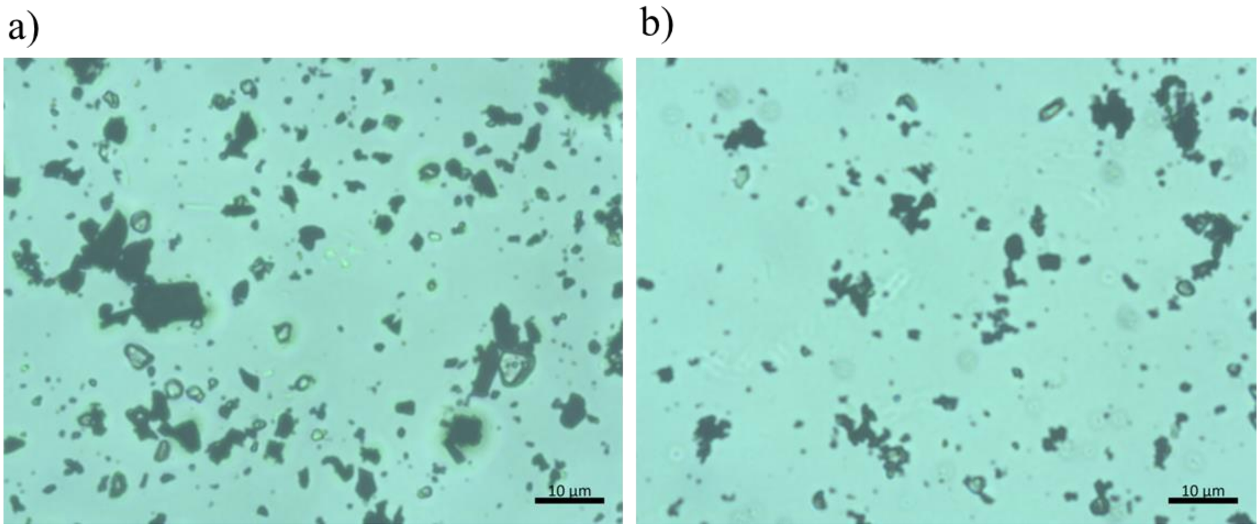

Figure S4: Optical microscope images of 10 wt% in kerosene MnB_2 a) Pre- and b) Post-Sonication for 2 hours. Post-sonicated samples possess a finer grain size and more even distribution.

References

[1] Evgeny Epifanovsky, Andrew T. B. Gilbert, Xintian Feng, Joonho Lee, Yuezhi Mao, Narbe Mardirossian, Pavel Pokhilko, Alec F. White, Marc P. Coons, Adrian L. Dempwolff, Zhengting Gan, Diptarka Hait, Paul R. Horn, Leif D. Jacobson, Ilya Kaliman, Jörg Kussmann, Adrian W. Lange, Ka Un Lao, Daniel S. Levine, Jie Liu, Simon C. McKenzie, Adrian F. Morrison, Kaushik D. Nanda, Felix Plasser, Dirk R. Rehn, Marta L. Vidal, Zhi-Qiang You, Ying Zhu, Bushra Alam, Benjamin J. Albrecht, Abdulrahman Aldossary, Ethan Alguire, Josefine H. Andersen, Vishikh Athavale, Dennis Barton, Khadiza Begam, Andrew Behn, Nicole Bellonzi, Yves A. Bernard, Eric J. Berquist, Hugh G. A. Burton, Abel Carreras, Kevin Carter-Fenk, Romit Chakraborty, Alan D. Chien, Kristina D. Closser, Vale Cofer-Shabica, Saswata Dasgupta, Marc de Wergifosse, Jia Deng, Michael Diedenhofen, Hainam Do, Sebastian Ehlert, Po-Tung Fang, Shervin Fatehi, Qingguo Feng, Triet Friedhoff, James Gayvert, Qinghui Ge, Gergely Gidofalvi, Matthew Goldey, Joe Gomes, Cristina E. González-Espinoza, Sahil Gulania, Anastasia O. Gunina, Magnus W. D. Hanson-Heine, Phillip H. P. Harbach, Andreas Hauser, Michael F. Herbst, Mario Hernández Vera, Manuel Hodecker, Zachary C. Holden, Shannon Houck, Xunkun Huang, Kerwin Hui, Bang C. Huynh, Maxim Ivanov, Ádám Jász, Hyunjun Ji, Hanjie Jiang, Benjamin Kaduk, Sven Kähler, Kirill Khistyaev, Jaehoon Kim, Gergely Kis, Phil Klunzinger, Zsuzsanna Koczor-Benda, Joong Hoon Koh, Dimitri Kosenkov, Laura Koulias, Tim Kowalczyk, Caroline M. Krauter, Karl Kue, Alexander Kunitsa, Thomas Kus, István Ladjánszki, Arie Landau, Keith V. Lawler, Daniel Lefrancois, Susi Lehtola, Run R. Li, Yi-Pei Li, Jiashu Liang, Marcus Liebenthal, Hung-Hsuan Lin, You-Sheng Lin, Fenglai Liu, Kuan-Yu Liu, Matthias Loipersberger, Arne Luenser, Aaditya Manjanath, Prashant Manohar, Erum Mansoor, Sam F. Manzer, Shan-Ping Mao, Aleksandr V. Marenich, Thomas Markovich, Stephen Mason, Simon A. Maurer, Peter F. McLaughlin, Maximilian F. S. J. Menger, Jan-Michael Mewes, Stefanie A. Mewes, Pierpaolo Morgante, J. Wayne Mullinax, Katherine J. Oosterbaan, Garrette Paran, Alexander C. Paul, Suranjan K. Paul, Fabijan Pavošević, Zheng Pei, Stefan Prager, Emil I. Proynov, Ádám Rák, Eloy Ramos-Cordoba, Bhaskar Rana, Alan E. Rask, Adam Rettig, Ryan M. Richard, Fazle Rob, Elliot Rossomme, Tarek Scheele, Maximilian Scheurer, Matthias Schneider, Nickolai Sergueev, Shaama M. Sharada, Wojciech Skomorowski, David W. Small, Christopher J. Stein, Yu-Chuan Su, Eric J. Sundstrom, Zhen Tao, Jonathan Thirman, Gábor J. Tornai, Takashi Tsuchimochi, Norm M. Tubman, Srimukh Prasad Veccham, Oleg Vydrov, Jan Wenzel, Jon Witte, Atsushi Yamada, Kun Yao, Sina Yeganeh, Shane R. Yost, Alexander Zech, Igor Ying Zhang, Xing Zhang, Yu Zhang, Dmitry Zuev, Alán Aspuru-Guzik, Alexis T. Bell, Nicholas A. Besley, Ksenia B. Bravaya, Bernard R. Brooks, David Casanova, Jeng-Da Chai, Sonia Coriani, Christopher J. Cramer, György Cserey, A. Eugene DePrince III, Robert A. DiStasio Jr., Andreas Dreuw, Barry D. Dunietz, Thomas R. Furlani, William A. Goddard III, Sharon Hammes-Schiffer, Teresa Head-Gordon, Warren J. Hehre, Chao-Ping Hsu, Thomas-C. Jagau, Yousung Jung, Andreas Klamt, Jing Kong, Daniel S. Lambrecht, WanZhen Liang, Nicholas J. Mayhall, C. William McCurdy, Jeffrey B. Neaton, Christian Ochsenfeld, John A. Parkhill, Roberto Peverati, Vitaly A. Rassolov, Yihan Shao, Lyudmila V. Slipchenko, Tim Stauch, Ryan P. Steele, Joseph E. Subotnik, Alex J. W. Thom, Alexandre Tkatchenko, Donald G. Truhlar, Troy Van Voorhis, Tomasz A. Wesolowski, K. Birgitta Whaley, H. Lee Woodcock III, Paul M. Zimmerman, Shirin Faraji, Peter M. W. Gill,

Martin Head-Gordon, John M. Herbert, and Anna I. Krylov. *Software for the frontiers of quantum chemistry: An overview of developments in the Q-Chem 5 package.* [*J. Chem. Phys.*. 155, 084801 (2021)]ss

[2] Y. Zhang and W. Yang, Phys. Rev. Lett. 80, 890 1998.

[3] P. J. Hay and R. L. Martin (1998) J. Chem. Phys. 109, pp. 3875.

[4] P. J. Hay and W. R. Wadt (1985a) J. Chem. Phys. 82, pp. 270.

[5] P. J. Hay and W. R. Wadt (1985b) J. Chem. Phys. 82, pp. 299.

[6] P. J. Hay (1983) J. Chem. Phys. 79, pp. 5469.

[7] W. R. Wadt and P. J. Hay (1985) J. Chem. Phys. 82, pp. 284.

[8] J. S. Binkley, J. A. Pople, and W. J. Hehre (1980) J. Am. Chem. Soc. 102, pp. 939
